# Characterization and selective incorporation of small non-coding RNAs in non-small cell lung cancer extracellular vesicles

**DOI:** 10.1186/s13578-018-0202-x

**Published:** 2018-01-10

**Authors:** Chuang Li, Fang Qin, Fen Hu, Hui Xu, Guihong Sun, Guang Han, Tao Wang, Mingxiong Guo

**Affiliations:** 1 Hubei Key Laboratory of Cell Homeostasis, College of Life Sciences, Wuhan University, Wuhan, 430072 Hubei People’s Republic of China; 2 Department of Respiratory and Critical Care Medicine, Tongji Hospital, Tongji Medical College, Huazhong University of Science and Technology, 1095 Jiefang Avenue, Wuhan, 430030 Hubei People’s Republic of China; 3 School of Basic Medical Sciences, Wuhan University, Wuhan, 430071 Hubei People’s Republic of China; 4 Department of Radiation Oncology, Hubei Cancer Hospital, 116 Zhuodaoquan South Road, Wuhan, 430079 Hubei People’s Republic of China; 5 Department of Oncology, Renmin Hospital of Wuhan University, 99 Zhangzhidong Street, Wuhan, 430060 Hubei People’s Republic of China

**Keywords:** Small non-coding RNAs, Extracellular vesicle, NSCLC, YRNAs, miRNAs

## Abstract

**Background:**

Extracellular vesicles (EVs) play important roles in intercellular communication through the delivery of their cargoes, which include proteins, lipids, and RNAs. Increasingly, multiple studies have reported the association between EV small non-coding RNAs and cancer, due to their regulatory functions in gene expression. Hence, analysis of the features of small non-coding RNA expression and their incorporation into EVs is important for cancer research.

**Results:**

We performed deep sequencing to investigate the expression of small RNAs in plasma EVs from lung adenocarcinoma (ADC) patients, lung squamous cell carcinoma (SQCC) patients, and healthy controls. Then, eighteen differently expressed miRNAs in plasma EVs was validated by QRT-PCR. The small RNA expression profiles of plasma EVs were different among lung ADC, SQCC patients, and healthy controls. And many small RNAs, including 5′ YRNA hY4-derived fragments, miR-451a, miR-122-5p, miR-20a-5p, miR-20b-5p, miR-30b-5p, and miR-665, were significantly upregulated in non-small cell lung cancer (NSCLC) EVs. And the cell viability assays indicated that hY4-derived fragments inhibited the proliferation of lung cancer cell A549. By comparing the cellular and EV expression levels of six miRNAs in NSCLC cells, we found that miR-451a and miR-122-5p were significantly downregulated in NSCLC cell lysates, while significantly upregulated in NSCLC EVs.

**Conclusions:**

The differently expressed EV small RNAs may serve as potential circulating biomarkers for the diagnosis of NSCLC. Particularly, YRNA hY4-derived fragments can serve as a novel class of biomarkers, which function as tumor suppressors in NSCLC. Additionally, miR-451a and miR-122-5p may be sorted into NSCLC EVs in a selective manner.

**Electronic supplementary material:**

The online version of this article (10.1186/s13578-018-0202-x) contains supplementary material, which is available to authorized users.

## Background

Extracellular vesicles (EVs) are membrane surrounded structures released by cells, and contain complex cargoes including nucleic acid, protein, and lipids [1]. EVs are heterogeneous in size and intracellular origins, and the main two subtypes of EVs are exosomes and microvesicles. Exosomes are small EVs (< 150 nm) released through endosomal pathway, and microvesicles are 100–1000 nm vesicles budding from plasma membrane. Importantly, EVs, especially exosomes, are secreted by almost all types of cells in culture, and are abundant in various body fluids including blood, saliva, urine, and breast milk [2]. It is widely confirmed that exosomes are originally generated by endocytosis and persist in late endosomes; invagination of the limiting late endosomal membrane results in the formation multivesicular bodies (MVBs), from which exosomes are finally released into extracellular space upon fusing of MVBs and the cell membrane [3]. Exosomes play important roles in intercellular communication, through direct interaction of exosomal proteins with signaling receptors of target cells [4], or by delivery of their contents into the recipient cell cytosol following fusion with recipient cell membranes through endocytic pathways [5]. Other studies have demonstrated that exosomes are involved in cancer initiation, progression, and metastasis [6]. Among the many types of molecules carried in exosomes, small RNAs, especially miRNAs, have attracted the most attention due to their roles in gene expression regulation.

Small RNAs are small (< 200 nucleotides) non-coding RNA molecules that include microRNA (miRNA), small nuclear RNA (snRNA), small nucleolar RNA (snoRNA), piwi-interacting RNA (piRNA), transfer RNA (tRNA), small ribosome RNA (rRNA), and small cytoplasmic RNA (Y RNA). These specialized RNAs play important roles in many different biological processes, such as RNA silencing, transcriptional regulation, chromosome replication, RNA processing, and regulation of protein trafficking and degradation [7]. It has increasingly become clear that non-coding RNAs can be sorted into exosomes, protected from RNase degradation, and internalized by neighboring or distant cells, where they subsequently modulate cellular processes [8].

Multiple EV small RNAs play important roles in tumorigenesis; however, the function of small RNAs in tumor-derived EV is complicated. For example, tumor-derived exosomes can transfer oncogenic small RNAs to recipient cells, and promote proliferation, migration, angiogenesis, and extracellular matrix remodeling, thus promoting the formation of a premetastatic niche that contributes to tumor metastasis [9]. In contrast, cancer cell-derived exosomes can mediate secretion of tumor suppressor small RNAs during tumor progression as a mechanism of coordinately suppressing tumor metastasis [10, 11].

Based on their cancer-specific expression profiles and the ability to detect them non-invasively in patient blood, small non-coding RNA molecules are promising biomarkers for cancer diagnosis [12, 13]. For example, circulating exosomal snRNA RNU6-1 alone or in combination with miR-320/miR-574-3p/RNU6-1 is a potential biomarker for glioblastoma multiforme [14], and exosomal miR-21 is a diagnostic biomarker for breast cancer, hepatocellular carcinoma, and esophageal squamous cell carcinoma [15–17]. In pancreatic ductal adenocarcinoma, the exosomal miRNA signature is more diagnostically relevant than exosomal protein glypican-1 levels [18], illustrating the potential advantages of exosomal small RNAs as biomarkers for cancer diagnosis.

Lung cancer is the leading worldwide cause of cancer-related death for both men and women [19]. The two main pathological types of lung cancer are NSCLC and small cell lung cancer (SCLC). Approximately 85–90% of all lung cancers are categorized as NSCLC [20], and the most common pathological types of NSCLC are adenocarcinoma (ADC; 30–50%) and squamous cell carcinoma (SQCC; 30%) [21]. In China in 2015, there were an estimated 733,300 new cases and 610,200 deaths due to lung cancer [22]. Similarly, in the USA, the average 5-year survival rate for lung cancer is only 18%, and more than one-half of lung cancer patients are diagnosed at a late stage, for which the 5-year survival rate is as low as 4% due to lack of effective treatments [19, 23]. Hence, investigating the genetic differences between NSCLC and healthy patients is important for understanding the pathogenesis of NSCLC and discovery of novel diagnostic biomarkers and therapeutic targets.

Adenocarcinoma and SQCC have clinical and pathological differences [24], and in order to identify specific diagnostic biomarkers or treatment targets for NSCLC subtypes, researchers have focused on genetic alterations or gene expression differences between lung ADC and SQCC [25, 26]. Importantly, although miRNA expression profiles are available, there is little information about the global expression differences of small non-coding RNAs in lung ADC and SQCC.

In recent years, it has become clear that exosomal miRNAs are closely associated with lung cancer. For example, miR-21, miR-155, miR-200b, and miR-379, are abnormally expressed in lung cancer-derived exosomes [27, 28], and tumor-derived exosomal miRNAs have shown promise as biomarkers for lung cancer subtypes, including ADC-specific miRNAs miR-181-5p, miR-30a-3p, miR-30e-3p, and miR-361-5p, as well as SQCC-specific miRNAs miR-10b-5p, miR-15b-5p and miR-320b [29]. Importantly, however, a comprehensive analysis and extensive verification of circulating exosomal small RNAs in NSCLC subtypes is still necessary for further understanding of NSCLC pathogenesis.

In the present study, we performed high-throughput sequencing of small RNAs in plasma EVs from lung ADC patients, SQCC patients, and healthy controls (CTRL). We found that a variety of small non-coding RNA species are present in plasma EVs, and EV small RNA fragments are likely to be associated with NSCLC pathogenesis. YRNA hY4-derived fragments were significantly upregulated in plasma EVs from both ADC and SQCC patients, and may be promising biomarkers for NSCLC diagnosis. Furthermore, we identified that the *RNY4P7* gene on chromosome 2 may not be a pseudogene, because the corresponding transcript detected in EVs and cells. Additionally, analysis of miRNAs demonstrated clear EV miRNA expression profile differences between ADC, SQCC, and CTRL groups, and suggested that certain miRNAs might be selectively sorted into NSCLC EVs.

## Results

### Characterization and properties of plasma EVs

To explore the expression profiles of EV small RNA in plasma from NSCLC patients, we collected the peripheral blood from patients with lung ADC, SQCC, or healthy controls (CTRL) (Table 1). All patients were characterized by clinical stage, and peripheral blood samples were collected before treatment. Clinical information, including diagnostic age and gender distribution are shown in Table 1.

**Table 1:** Clinical characteristics of plasma EV samples from NSCLC patients and healthy controls

	RNA-Seq			qRT-PCR		
	ADC	SQCC	CTRL	ADC	SQCC	CTRL
Number	28	13	13	37 (21)	27 (9)	16 (12)
Age of diagnosis (year, mean ± SD)	60.04 ± 7.89	63.08 ± 7.19	62.25 ± 10.21	60.15 ± 8.11	62.30 ± 7.68	53.38 ± 18.09
Male	16	12	12	23	26	15
Female	12	1	1	14	1	1

The numbers in brackets represent the numbers of samples contained in the RNA-Seq group

*ADC* adenocarcinoma, *SQCC* squamous cell carcinoma, *CTRL* healthy controls

**Fig. 1:**
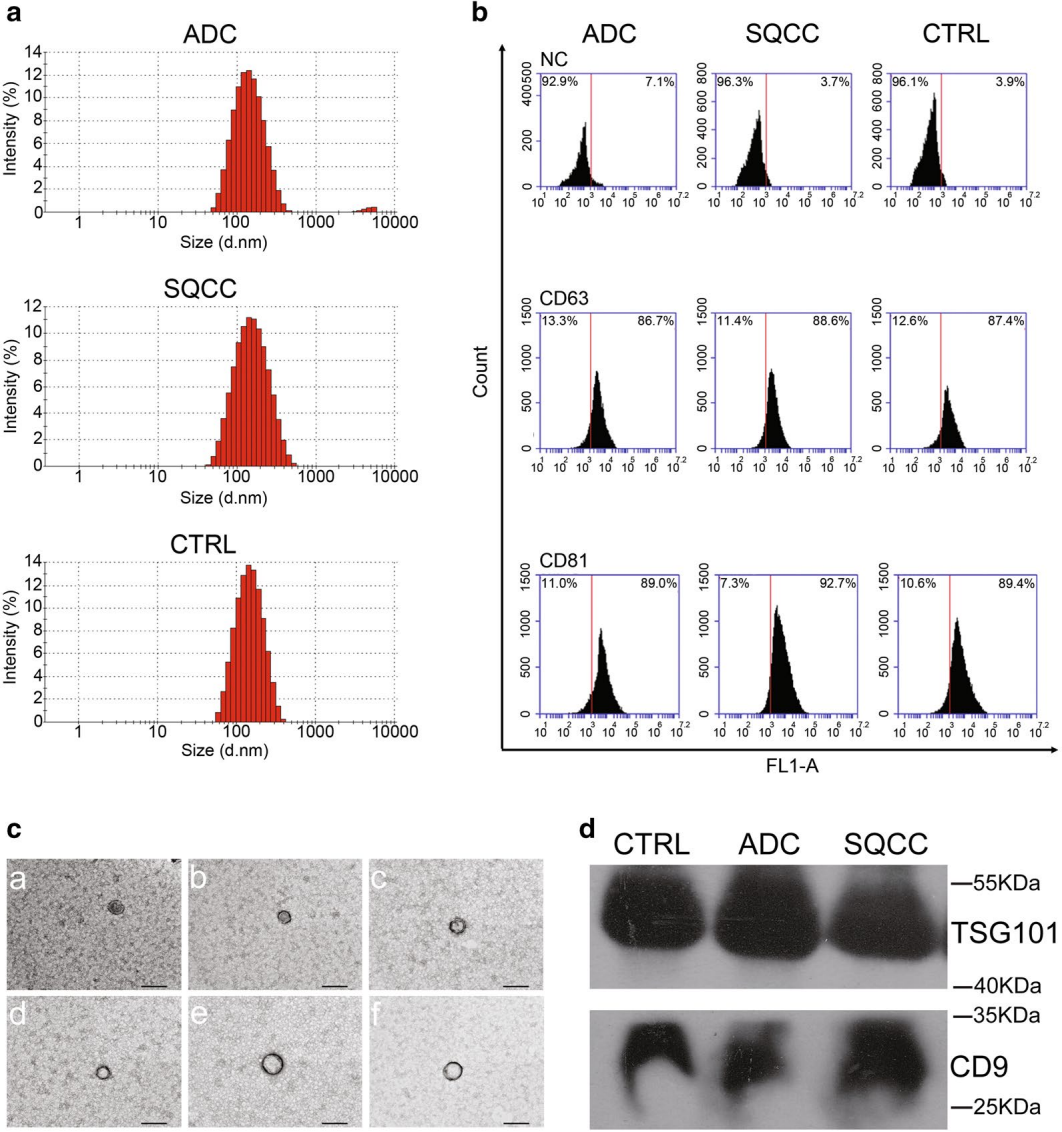
Characterization of EVs from plasma samples of NSCLC patients and controls. **a** Size distribution of plasma EVs by nanoparticle tracking analysis. **b** Plasma EVs were analyzed by flow cytometry for the exosomal markers antibodies CD63 and CD81. **c** Negative staining TEM (× 40,000) of plasma EVs. The diverse microscopic fields are shown in (*a*–*f*), and the black solid lines represent 200 nm scale bars. **d** Western blot analysis of plasma EVs with exosomal markers antibodies TSG101 and CD9. Original uncropped Western blot images were reported in Additional file 1: Figure S7

Next, plasma EVs were extracted and the containing RNA was isolated for small RNA sequencing. The isolated EVs were analyzed by nanoparticle tracking analysis, transmission electron microscope (TEM), flow cytometry, and western blot. The nanoparticle tracking analysis (NTA) (Fig. 1a, Table 2) showed that the isolated EV fractions were mainly composed of particles in the acceptable size range for EVs from 50 to 400 nm, and also showed that the three EV isolates had a similar size distribution and peak region (100–200 nm). The NTA results also indicated that the isolated EVs were mainly composed by small EVs, namely exosomes. We also used flow cytometry analysis (Fig. 1b) to validate EVs with the generally accepted exosomal markers CD63 and CD81 [30], which were significantly positive (> 85%) in all EV groups. Negative staining TEM (Fig. 1c) of plasma EVs also illustrated a typical diameter of 30–120 nm and bilayer membrane structure. EVs were further confirmed by western blot analysis (Fig. 1d) to ensure expression of the exosomal markers, TSG101 and CD9 [31]. The above detections of exosomal markers suggested that the isolated EVs contained abundant exosomes. Together, these results demonstrate the reliability of isolating and characterizing human plasma EVs.

**Table 2:** Characterization of EV particle diameter

Sample	ADC	SQCC	CTRL
Average of particle diameter (nm)	124.1	126.6	126
Polydispersity index (PDI)^a^	0.229	0.235	0.198
Major peak of particle diameter (nm)	154.9	170.7	156.9
Percentage of 20–200 nm diameter (%)	78.3	71.8	79.4

*ADC* adenocarcinoma, *SQCC* squamous cell carcinoma, *CTRL* healthy controls

^a^Polydispersity index (PDI) is a dimensionless value that represents the distribution of particle size. PDI values of 0.08–0.7 indicate moderate dispersion system and optimum application scope of algorithm

### Small RNA expression profiles are different between NSCLC plasma EVs and controls

**Fig. 2:**
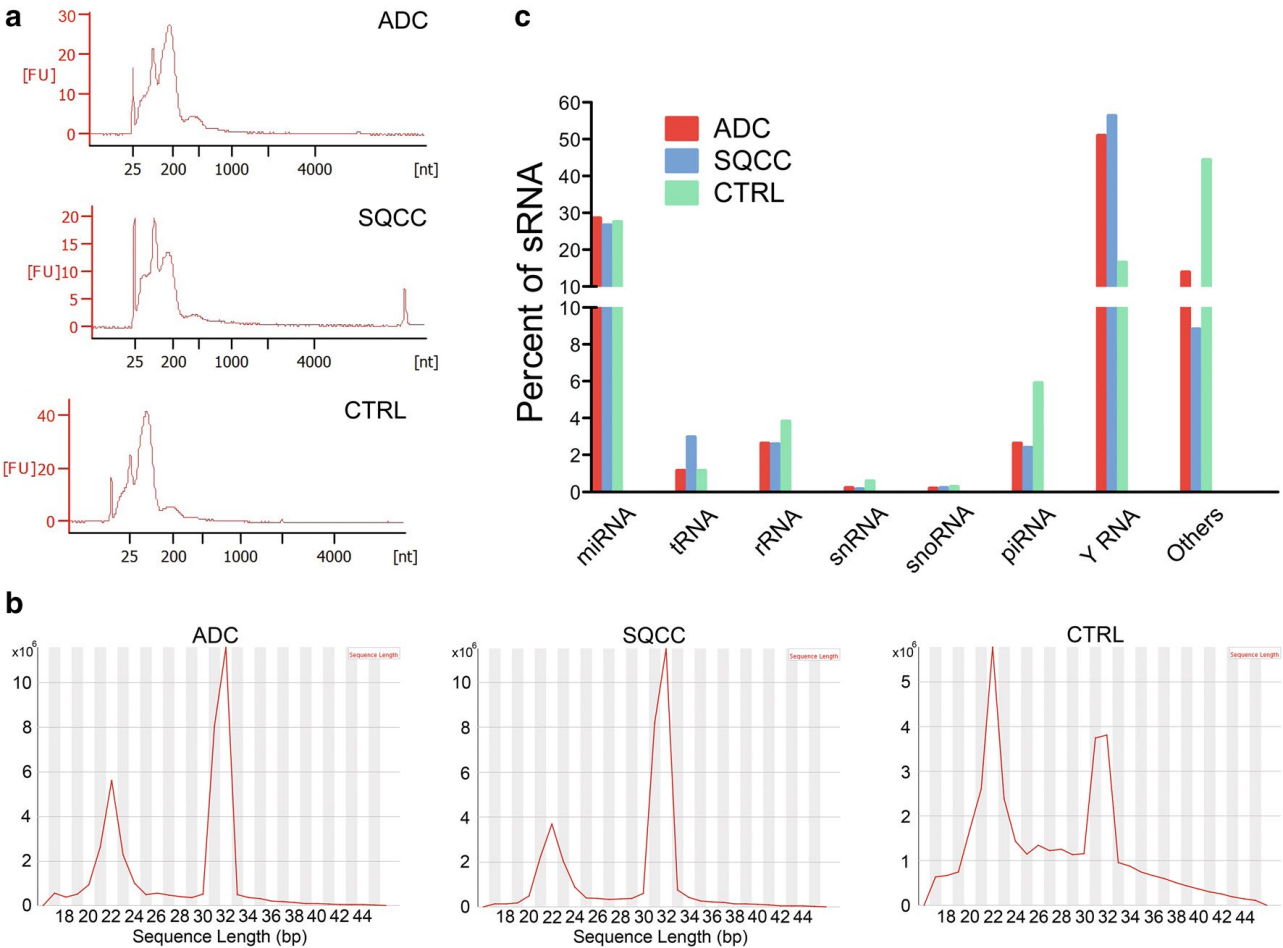
Characterization of plasma EV RNA profiles from NSCLC patients and controls. **a** Bioanalyzer electropherogram of total EV RNA from ADC, SQCC, and CTRL groups. These images are representative of two replicates. **b** Sequence length distribution analysis of small RNA from ADC, SQCC, and CTRL EVs by RNA-Seq. **c** Sequence reads uniquely aligned to the human genome were annotated to known small non-coding RNA transcripts. Data represents the percentage of the different types of small RNA in plasma EVs of ADC, SQCC, and CTRL groups

To evaluate the small RNA expression profiles from EVs, we isolated total EV RNA from ADC patients (28 samples), SQCC patients (13 samples), and healthy controls (13 samples). The RNA samples from same group were pooled together in a fixed ratio. Bioanalyzer RNA profiles of the three EV groups showed small RNA peaks at around 25, 25–200, and 200 nt, and the absence of 18S and 28S rRNA peaks (Fig. 2a). This is consistent with previous reports demonstrating that plasma exosomal RNAs are mainly small RNAs, with little or no 18S and 28S rRNA [32, 33]. Additionally, these results also suggest that EVs from the three sample groups contain different small RNA signatures. For instance, CTRL EV had a small RNA peak at less than 20 nt that is absent from the other groups, as well as a 200 nt small RNA peak that is lower than the other groups.

Small RNA fragments (17–45 nt) were then processed for analysis by high-throughput sequencing. After filtering the raw data, a total of 38,691,242, 34,607,916, and 37,041,034 clean reads were obtained for ADC, SQCC, and CTRL groups, respectively (Table 3). We mapped 95.1817, 95.4355, and 93.9549% of clean reads to the human genome (hg19) for ADC, SQCC, and CTRL, respectively. The distribution of small RNA sequence length over all clean reads showed that all EV small RNAs had common peaks at 18–24 and 30–33 nt; of interest, the 30–33 nt small RNA peak in the CTRL group was threefold lower than that in ADC and SQCC groups (Fig. 2b), which suggests that 30–33 nt small RNAs were highly expressed in NSCLC plasma EVs.

**Table 3:** The data filtering statistics of RNA-Seq data from NSCLC and control EVs

Type	ADC		SQCC		CTRL	
	Count	Percent (%)	Count	Percent (%)	Count	Percent (%)
Total reads	41,609,790	100	36,662,475	100	45,042,122	100
3′adapter NULL	796,544	1.91	736,542	2.01	923,352	2.05
Insert NULL	406,921	0.98	97,794	0.27	4,748,412	10.54
5′adapter contaminants	23,913	0.06	10,567	0.03	178,076	0.40
Removed inferior quality	202,479	0.49	169,470	0.46	268,256	0.60
Smaller than 17 nt	1,482,425	3.56	1,033,392	2.82	1,843,389	4.09
Poly-A/T/C/G/N	6266	0.02	6794	0.02	39,603	0.09
Clean reads	38,691,242	92.99	34,607,916	94.40	37,041,034	82.24

The RNA-Seq filtering data of plasma EVs are shown above as a count and percentage

*ADC* adenocarcinoma, *SQCC* squamous cell carcinoma, *CTRL* healthy controls

**Fig. 3:**
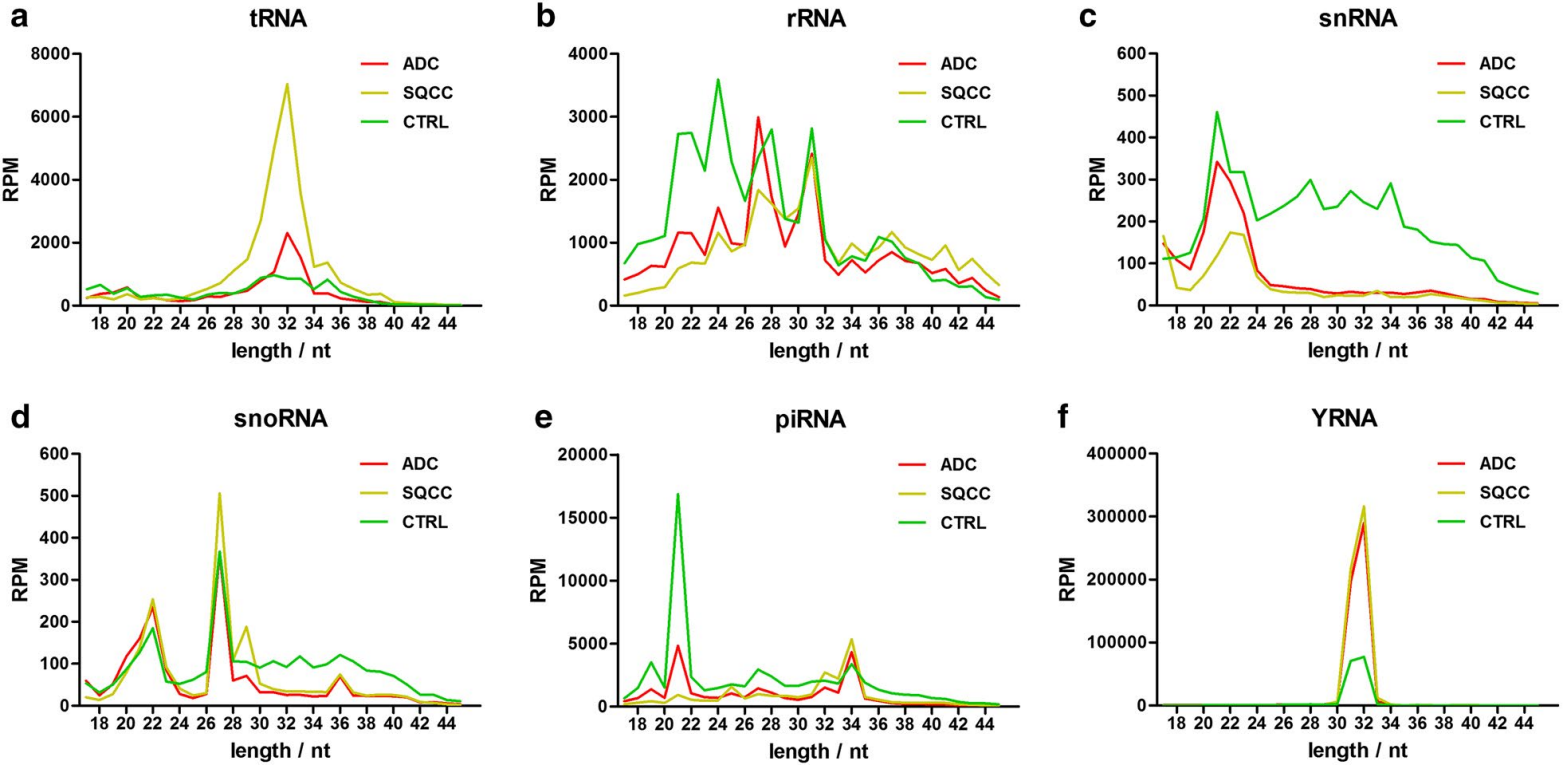
The size distribution and relative abundance of various small RNA in plasma EVs shows diversity among ADC, SQCC, and CTRL groups. The relative abundance of tRNA (**a**), rRNA (**b**), snRNA (**c**), snoRNA (**d**), piRNA (**e**), and YRNA (**f**) were normalized as RPM (number of mapped reads per million clean reads) and shown respectively

We then categorized and annotated the uniquely aligned sequence reads to known non-coding small RNA transcripts, and found that the majority of reads corresponded to YRNA fragments, miRNA, and other unknown small RNA species (Fig. 2c). We found that the total abundance of YRNA fragments in plasma EVs from ADC (50.92%) and SQCC (56.29%) was remarkably higher than that from CTRL (16.51%). Additionally, piRNA, rRNA, snRNA, and other unknown small RNA fragments were lower in the ADC and SQCC groups than in the CTRL group, and tRNA fragments from SQCC plasma EVs (2.95%) were higher than that from the ADC (1.13%) and CTRL (1.13%) groups. When we analyzed the length distribution of plasma EV small RNAs, we found that the variety of small RNAs (such as YRNA, tRNA, piRNA, rRNA, and snRNA) among the three sample groups had distinctly different length distribution and abundance (Fig. 3).

All sample groups had a major tRNA fragment peak at 29–34 nt, but the abundance of tRNA fragments in the SQCC group was about threefold higher than in ADC and CTRL groups (Fig. 3a). The reads for rRNA fragments in all sample groups were mainly distributed at 20–32 nt, and the RPM of the CTRL group at 20–26 nt was higher than ADC and SQCC (Fig. 3b). The snRNA fragment reads were mainly distributed at 19–24 nt, and the RPM of the CTRL group was higher than in the ADC and SQCC groups at the longer snRNA fragment (> 24 nt) (Fig. 3c). We found no significant difference in snoRNA fragments at the major peak (about 27 nt) or minor peak (18–24 nt), but the RPM of the CTRL group was slightly higher than ADC and SQCC at 30–42 nt (Fig. 3d). In addition, the RPM for piRNA fragments in the CTRL group was significantly higher than in the ADC and SQCC groups at the major peak of 20–22 nt (Fig. 3e). All groups had a main YRNA peak at 30–33 nt, and the YRNA fragment abundance in ADC and SQCC groups at the main peak was significantly higher than CTRL (Fig. 3f). Together, our initial data indicates that the expression signature of EV small RNA species is different in NSCLC and healthy samples.

### hY4 RNA fragments are upregulated in NSCLC EVs and inhibit NSCLC cell proliferation

**Fig. 4:**
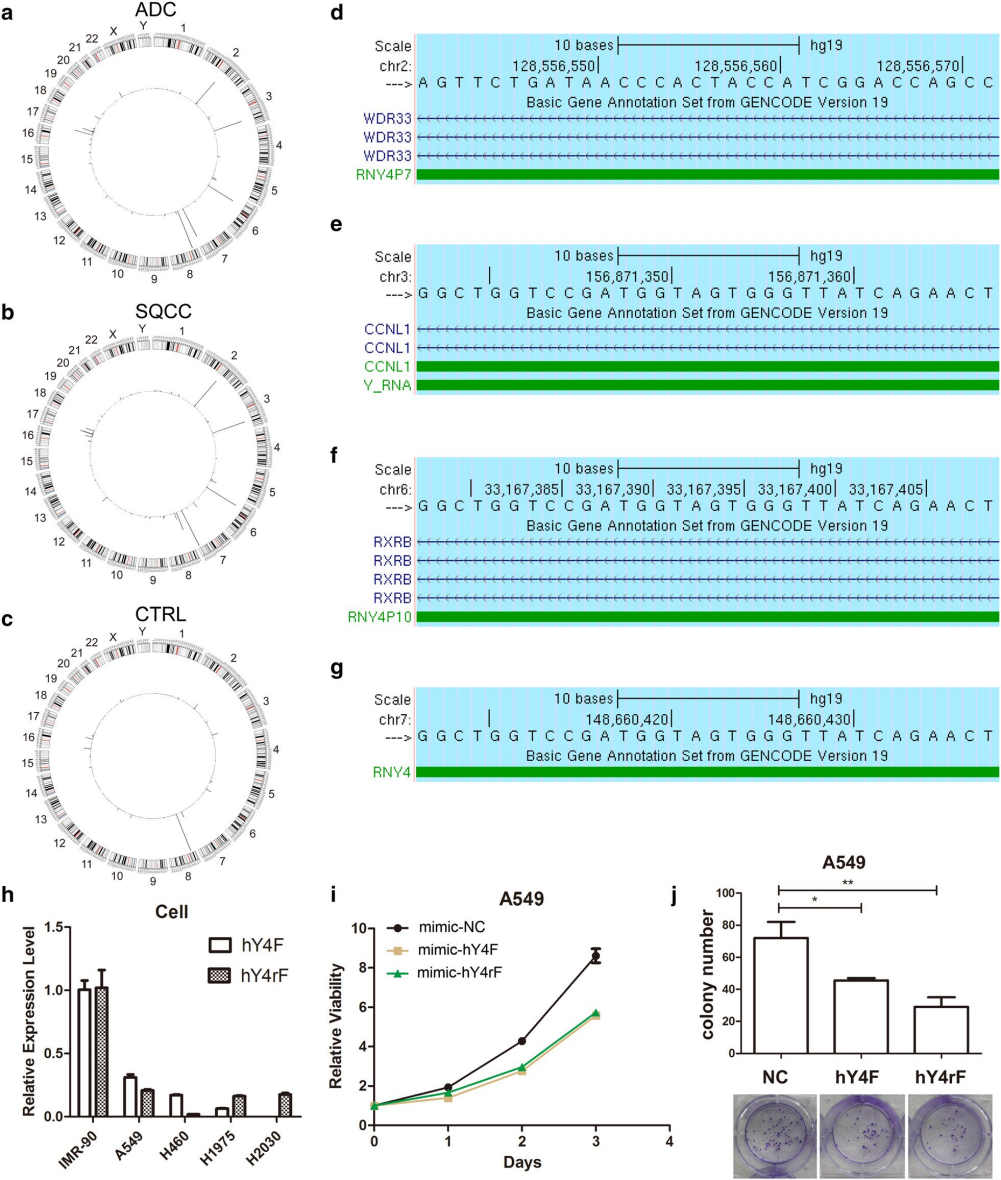
hY4 RNA-derived fragments are upregulated in NSCLC EVs, and may be selectively sorted into NSCLC-derived EVs. The Circos plot shows the distribution of small RNAs from ADC (**a**), SQCC (**b**), and CTRL (**c**) EVs. The outer circle represents the chromosome band, and the line segment on the inner circle represents the abundance of mapped sequences. **d**–**g** Sequence alignment of the genomic (hg19) location of the hY4 fragment (hY4F) and hY4 reverse fragment (hY4rF) from the UCSC Genome Browser. **h** The expression of hY4F and hY4rF in NSCLC-derived EVs and cell lysates compared to control IMR-90 cells (U6 snRNA was used as an internal control). **i** MTT assay for overexpression of hY4F and hY4rF mimics in A549 cells. **j** Colony formation assay for A549 cells treated with hY4F and hY4rF mimics (representative photograph was shown below the column). *P < 0.05, **P < 0.01. Results are presented as mean ± SD

To investigate the specific types and sequences of YRNA fragments that are upregulated in NSCLC plasma EVs, we analyzed the distribution of corresponding genes from the human genome (hg19) (Fig. 4a–c). We found that the most abundant small RNA sequences mapped to loci on human chromosomes 2, 3, 6, 7, and 8; the small RNAs that mapped to chromosome 2, 3, 6, and 7 had greater abundance in NSCLC EVs than CTRL EVs, while the small RNAs that mapped to chromosome 8 were downregulated in SQCC EVs.

We confirmed that a small RNA on chromosome 8 was miR-320a (TCGCCCTCTCAACCCAGCTTTT), which is consistent with EV miRNA expression profiles (Additional file 1: Table S1). Importantly, we found that miR-320a was the most abundant EV miRNA in our samples, and was significantly downregulated in SQCC EVs. The small RNAs that mapped to chromosomes 2, 3, 6, and 7 were confirmed as the hY4 fragment (hY4F, AGTTCTGATAACCCACTACCATCGGACCAGCC) and hY4 reverse fragment (hY4rF, GGCTGGTCCGATGGTAGTGGGTTATCAGAACT), which aligned to the 5′-end of the hY4 RNA sequence. hY4F mapped to the *Y_RNA* gene (predicted YRNA from the Rfam database) on chromosome 3, the *RNY4P10* pseudogene on chromosome 6, and the *RNY4* gene on chromosome 7. Additionally, hY4rF mapped to the *RNY4P7* pseudogene on chromosome 2 (Fig. 4d–g).

It is generally acknowledged that YRNA genes are clustered together at a single locus on human chromosome 7q36 [34], however, 966 pseudogenes derived from YRNAs have been characterized in the human genome [35]. Recent reports demonstrate that some hY4 sequences previously annotated as pseudogenes are transcribed, processed, and secreted, suggesting their pseudogene annotation may not be accurate [36]. Our results are consistent with this, as most of the upregulated YRNA fragments in NSCLC EVs were identified as hY4 fragments. In addition, our results suggest that *RNY4P7* may not be a hY4 RNA pseudogene, as hY4rF, which is fully aligned to *RNY4P7*, was detected in both NSCLC cells and normal cells (Fig. 4h).

Then, we detected expression of hY4 RNA fragments in NSCLC cells, and found that both hY4F and hY4rF were significantly down-regulated in all the NSCLC cells we tested (Fig. 4h). Furthermore, we found that overexpression of hY4F and hY4rF could significantly suppress proliferation of NSCLC cell A549 (Fig. 4i, j). The above results indicate that hY4 RNA fragments, including hY4F and hY4rF may function as tumor suppressors in NSCLC.

### miRNAs are differentially expressed in NSCLC plasma EVs

**Fig. 5:**
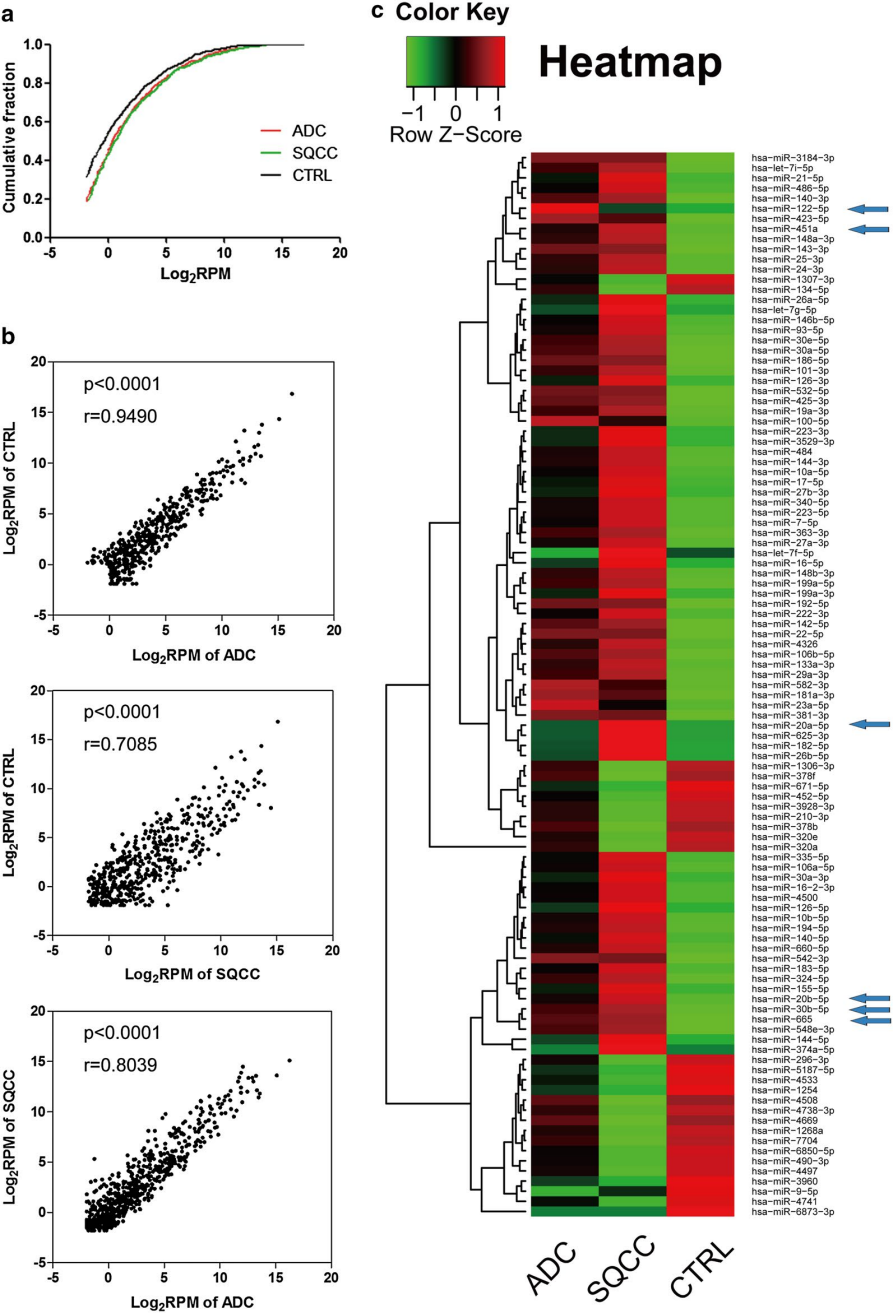
Comparative analysis of EV miRNA expression differences among ADC, SQCC, and CTRL groups. **a** Cumulative frequency distribution of the mean relative abundance of plasma EV miRNAs in ADC, SQCC, and CTRL groups. The mean relative abundance is shown as a Log2 scale (Log_2_ RPM), and the relative frequency is shown as a fraction. **b** Scatter plots showing the correlation between the expression of plasma EV miRNAs in the three groups (*p* P-value, *r* Pearson correlation coefficient). **c** Heatmap showing clustering and relative abundance of 105 miRNAs with significantly different expression (FDR < 0.05, P < 0.05, |log_2_FC| ≥ 1) between NSCLC plasma EVs and the control. The arrows show the miRNAs selected for qRT-PCR validation in cell lines

miRNAs are implicated in tumorigenesis across many cancer types. To determine whether miRNA species are differentially expressed in NSCLC EVs, clean sequencing reads were mapped to mature miRNA sequences in the miRBase database. Together, we identified 878 total miRNAs in ADC, SQCC, and CTRL plasma EVs (Additional file 1: Table S1). As shown in Fig. 5a, the cumulative frequency distribution curve of EV miRNAs detected in the CTRL group was higher than that in the ADC and SQCC groups, while the curves in the ADC and SQCC groups overlapped. Only 45.0% of miRNAs in ADC EVs and 43.5% of miRNAs in SQCC EVs had abundances less than 1 RPM, while 54.2% of detectable EV miRNAs in the CTRL group had abundances below that, suggesting that the majority of CTRL EV miRNAs have a relatively lower expression than ADC and SQCC mRNAs.

We next compared the expression of all miRNAs identified by small RNA sequencing to determine whether there were any correlations between different EV sample types. Overall, we found a strong positive correlation between ADC and CTRL (r = 0.9490), and a weaker correlation between ADC and SQCC (r = 0.8039), and SQCC and CTRL (r = 0.7085) (P values < 0.0001) (Fig. 5b). This data collectively suggests that while there are many differentially expressed miRNAs between the three groups, the majority of EV miRNAs have similar expression.

**Fig. 6:**
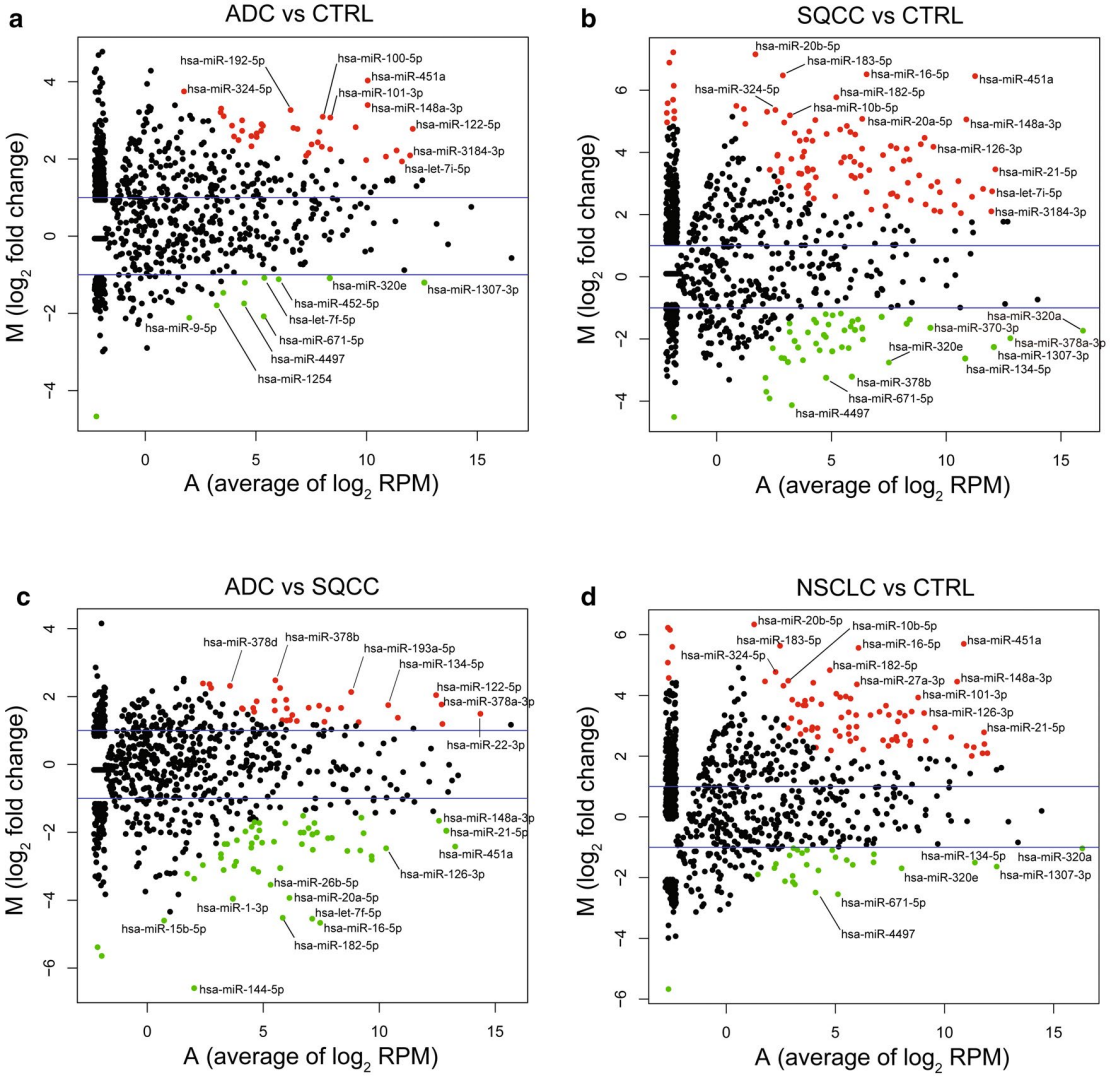
miRNA expression differences between the three experimental groups. MA plots showing differentially expressed miRNAs between ADC and CTRL (**a**), SQCC and CTRL (**b**), ADC and SQCC (**c**), and NSCLC (average of ADC and SQCC) and CTRL (**d**). The blue lines stand for |log_2_ FC| = 1, the red dots represent the significantly different miRNAs with over twofold upregulation, the green dots represent the significantly different miRNAs with over twofold downregulation, and the black dots represent miRNAs with no significant difference. FDR < 0.05, P < 0.05, and |log_2_FC| ≥ 1 was considered as statistically significant

To characterize miRNA expression signatures on a more global level, we first compared significant miRNA expression differences between NSCLC and CTRL EVs (Additional file 1: Figure S1), then performed clustering analysis. Because the abundance of many miRNAs is extremely low in EVs from all three groups, we excluded miRNAs with less than 1 RPM (Additional file 1: Figure S2 and Fig. 5c). MA plots were also generated, illustrating the M value (log_2_ Fold Change) and A value (average of log_2_ RPM) of miRNA expression from each of the three groups (Fig. 6). Together, these analyses demonstrate that there were distinct EV miRNAs expression profiles in ADC, SQCC, and CTRL groups.

The specific miRNAs that were expressed in plasma EVSs among ADC, SQCC patients, and CTRL group is shown in Additional file 1: Table S1, and the significantly changed miRNAs are listed in Additional file 1: Tables S2–S5. For ADC vs. CTRL, ADC EVs contained 37 miRNAs that were significantly upregulated, and 11 miRNAs that were significantly downregulated. For SQCC vs. CTRL, 98 miRNAs were significantly upregulated in SQCC, while 47 miRNAs were significantly downregulated. For NSCLC (average of ADC and SQCC) vs. CTRL, 78 miRNAs were significantly upregulated, while 27 miRNAs were significantly downregulated. For ADC vs. SQCC, 33 miRNAs were significantly upregulated in ADC EVS s, while 56 miRNAs were significantly downregulated. In summary, our data demonstrates that there are many distinct EV miRNA expression signatures in ADC and SQCC patients compared to CTRL group, which suggests that there may be certain miRNA candidates that may serve as specific biomarkers for either ADC and SQCC.

### qRT-PCR verification of miRNAs in NSCLC plasma EVs

**Fig. 7:**
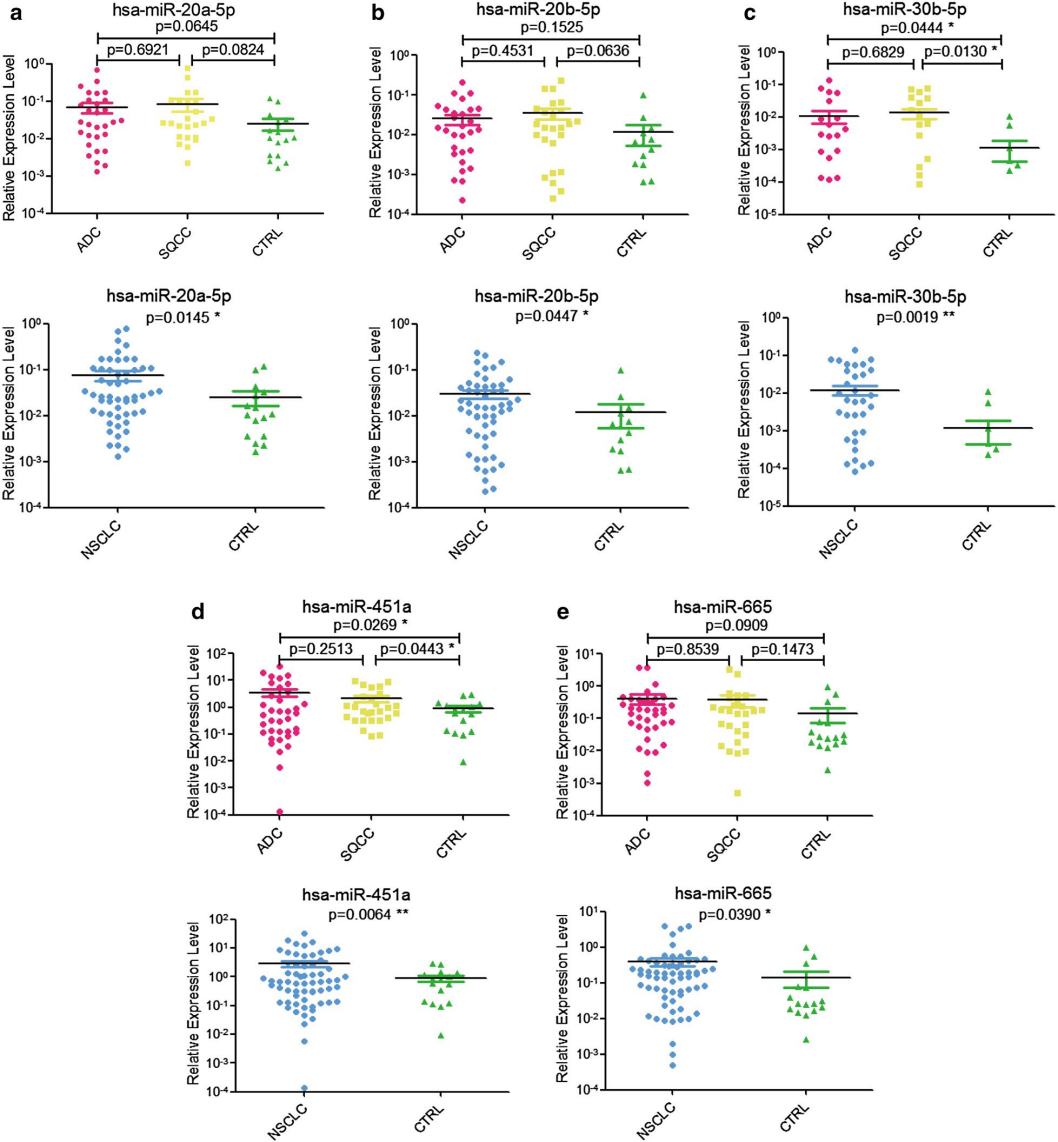
qRT-PCR analysis of miRNA expression in EVs from NSCLC patients and healthy controls. The qRT-PCR analysis of miR-20a-5p (**a**), miR-20b-5p (**b**), miR-30b-5p (**c**), miR-451a (**d**), and miR-665 (**e**) expression in EVs from ADC, SQCC, and CTRL, or between NSCLC and CTRL are shown. The qRT-PCR data were normalized to cel-miR-39. An unpaired Student’s t test was used for equal variances, and Welch’s t test was used for unequal variances. *P < 0.05, **P < 0.01. Results are presented as mean ± SEM

To further confirm the miRNA expression differences in plasma EVs from lung cancer patients and CTRL group, we validated candidate miRNAs using qRT-PCR in each plasma EV group, including 21 ADC, 9 SQCC, and 12 CTRL samples (Table 1). Eighteen miRNAs with top-ranking differences between NSCLC EVs and controls were selected for verification, including both high and low abundance miRNAs (Table 4). When we combined expression in ADC and SQCC exosomes into one NSCLC exosome group, we found that miR-20a-5p, miR-20b-5p, and miR-665 were significantly upregulated in NSCLC plasma EVs, while their expression was not significantly different among the single groups (Fig. 7a, b and e). Importantly, however, expression of miR-30b-5p and miR-451a was significantly greater in ADC and SQCC EVs than in CTRL EVs (Fig. 7c, d).

**Table 4:** RNA-Seq results of EV miRNAs selected for qRT-PCR detection

miRNA	ADC	SQCC	CTRL
hsa-miR-1307-3p	4109.3279	1974.5771	9459.7251
hsa-let-7f-5p	28.6111	668.7487	60.5545
hsa-miR-134-5p	2454.9742	731.1044	4519.9602
hsa-miR-451a	4285.621	22,923.1081	261.8717
hsa-miR-148a-3p	3435.5578	10,867.1669	326.0708
hsa-miR-192-5p	294.2785	329.231	30.5337
hsa-miR-122-5p	11,372.4961	2761.3914	1655.3534
hsa-miR-20a-5p	17.911	273.8969	9.503
hsa-miR-625-3p	18.4538	153.3464	12.0137
hsa-miR-324-5p	12.3542	37.7948	0.9179
hsa-miR-20b-5p	5.2725	38.4305	0.27
hsa-miR-144-5p	0.4135	39.8753	0
hsa-miR-30b-5p	6.7199	31.6402	0
hsa-miR-665	7.1592	18.7818	0
hsa-miR-374a-5p	0	12.6272	0
hsa-miR-183-5p	8.2189	69.6084	0.7829
hsa-miR-10b-5p	12.4318	55.2475	1.5118
hsa-miR-6715b-3p	4.8848	0	0

The expression of miRNAs was normalized as RPM

*ADC* adenocarcinoma, *SQCC* squamous cell carcinoma, *CTRL* healthy controls

Our above results indicate that expression of miR-20a-5p, miR-20b-5p, miR-30b-5p, miR-451a, and miR-665 are significantly upregulated in NSCLC plasma EVs, which is consistent with the RNA-Seq results. However, the expression of other 13 miRNAs tested by qRT-PCR was not significantly different among the three EV sample groups (Additional file 1: Figure S3), which was in contrast to our RNA-Seq results. These differences may be explained by individual variation of EV miRNAs due to biological heterogeneity [37] of lung tumors [38, 39].

### Enrichment of certain microRNAs in NSCLC EVs

**Fig. 8:**
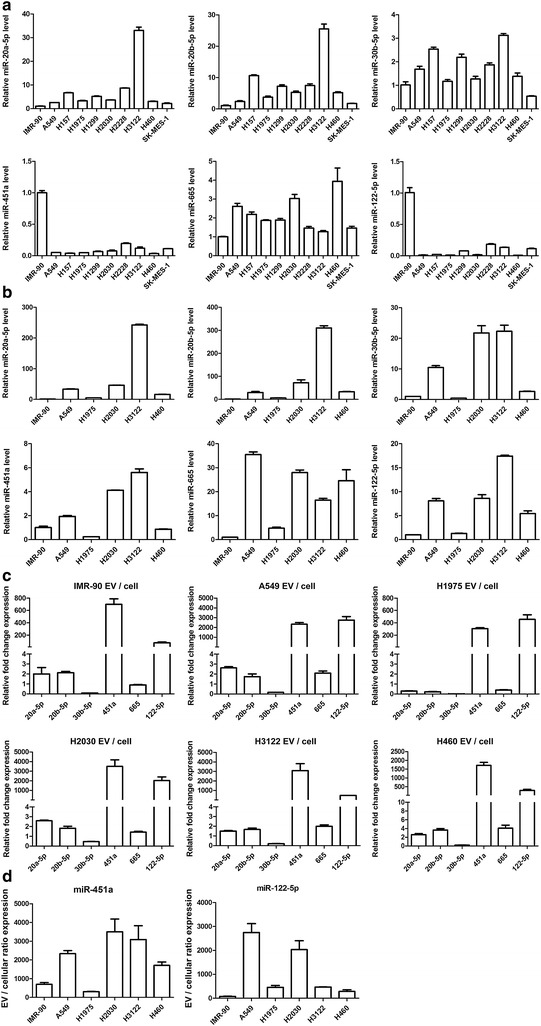
Some miRNAs are selectively enriched in EVs secreted from NSCLC cell lines. **a** qRT-PCR results showing the expression of miR-20a-5p, miR-20b-5p, miR-30b-5p, miR-451a, miR-665 and miR-122-5p in NSCLC cell lines and the normal control IMR-90 cell line. **b** qRT-PCR results showing the expression of the above miRNAs in EVs from the conditioned culture medium of NSCLC or IMR-90 cells. **c** The fold change of expression of the above miRNAs in EVs or cell lysates. **d** EV vs. cell lysate expression fold changes of miR-451a and miR-122-5p. The qRT-PCR data were normalized to cel-miR-39. Results are presented as mean ± SD

It has been previously reported that miR-451a [40] and miR-122-5p [41] are downregulated in NSCLC cells, yet we found they were upregulated in NSCLC EVs compared with healthy controls. Previous studies have indicated that some small non-coding RNAs, including specific miRNAs are sorted into exosomes in a selective manner [42], which may explain the discrepancy. To confirm whether specific miRNAs are sorted into NSCLC EVs in a selective manner, we compared the cellular and EV expression levels of six miRNAs (miR-451a, miR-122-5p, miR-20a-5p, miR-20b-5p, miR-30b-5p, and miR-665) in NSCLC cells compared to the normal embryonic lung fibroblast cell line IMR-90. As shown in Fig. 8a, miR-20a-5p, miR-20b-5p, miR-30b-5p, and miR-665 were upregulated in most NSCLC cells, while miR-451a and miR-122-5p were significantly downregulated.

Next, we compared the expression of these miRNAs in EVs from each cell line (Fig. 8b). We found that miR-451a, miR-122-5p, miR-20a-5p, miR-20b-5p, miR-30b-5p, and miR-665 were significantly upregulated in EVs from most of the tested NSCLC cells, consistent with our RNA-Seq and qRT-PCR results. Finally, we compared the cellular expression of these miRNAs to the matched EV expression for each cell line. As shown in Fig. 8c and d, miR-451a and miR-122-5p were significantly higher in EVs from all cell types compared to matched cells, suggesting that these miRNAs may be selectively sorted into NSCLC EVs. We also found that the EV vs. cellular ratio fold change expression of miR-451a in all NSCLC cell lines, except for H1975, was higher than in control cells (Fig. 8d), further indicated its potential to be selectively sorted into EVs.

### GO and KEGG analysis of differentially expressed miRNA

To investigate the potential biological functions and pathways in which differentially expressed NSCLC plasma EV miRNAs are involved, we performed GO (Gene Ontology) biological process enrichment analysis and KEGG (Kyoto Encyclopedia of Genes and Genomes) pathway enrichment analysis with the predicted miRNA targets. The GO analysis results (Additional file 1: Figure S4) showed that the predicted targets of miR-20a-5p, miR-20b-5p, miR-30b-5p, and miR-122-5p were similarly enriched for GO biological process terms, including regulation of macromolecule metabolic processes, regulation of nucleobase, nucleoside, nucleotide and nucleic acid metabolic processes, regulation of nitrogen compound metabolic processes, and regulation of macromolecule biosynthetic processes. However, we found that the GO enrichment results of predicted miR-451a and miR-665 targets were extremely different. The predicted targets of miR-451a were enriched in regulation of catalytic activity, regulation of cell proliferation, regulation of phosphate metabolism, and regulation of phosphorylation. In contrast, the predicted miR-665 targets had major enrichment in development-related biological processes, such as respiratory system development, nervous system development, lung development, respiratory tube development, blood vessel morphogenesis, and embryonic skeletal system development. Together, these results demonstrate that these EV miRNAs may have both common and specific functions, suggesting that complicated regulation networks exist among potential cancer-related EV miRNAs.

The KEGG results (Additional file 1: Figure S5) show that the predicted targets of some miRNAs were significantly enriched in common pathways. For example, the predicted targets of all analyzed miRNAs, except miR-665, were enriched for endocytosis (kegg: hsa04144), the predicted targets of miR-122-5p, miR-20a-5p, miR-20b-5p, miR-30b-5p, and miR-665 were enriched for cancer pathways (kegg: hsa05205), and miR-122-5p, miR-20a-5p, miR-20b-5p, and miR-665 were enriched for cancer proteoglycans (kegg: hsa05205). Additionally, the KEGG results showed that predicted targets of these miRNAs are enriched for multiple pathways involved in NSCLC, such as MAPK signaling (kegg: hsa04010), Wnt signaling (kegg: hsa04310), and FoxO signaling (kegg: hsa04068), suggesting that these miRNAs are likely to regulate signaling pathways involved in NSCLC pathogenesis.

Our study has demonstrated that miR-451a and miR-122-5p are selectively sorted into NSCLC EVs, and given the importance of the endocytic pathway in the selective sorting and uptake of EVs [5, 43], we analyzed the KEGG enrichment of predicted miR-451a and miR-122-5p targets in the endocytic pathway. As shown in Additional file 1: Figure S6, predicted targets of miR-451a and miR-122-5p are enriched for both clathrin-dependent and clathrin-independent endocytosis. In addition, some enriched targets of either miRNA are the same genes, or belong to the same gene families, including CAV1/CAV2, KIF5B/KIF5C, ARPC2/ARPC3, and GIT1/GIT2. Together, the above results indicate that miR-122-5p and miR-451a are likely to regulate the endocytic pathway by similar mechanisms.

## Discussion

Since the presence of miRNAs in EVs was published about 10 years ago [44], multiple types of small non-coding RNAs have been found in EVs [45], and many studies have indicated that some miRNAs are differentially expressed in cancer exosomes, and are closely related to tumorigenesis [8, 9]. Circulating EV small RNAs, especially miRNAs, are also potential non-invasive biomarkers for cancer diagnosis [13–15]. Overall, we have demonstrated that the small RNA expression profiles of plasma EVs are different among lung ADC, SQCC patients, and healthy controls. In particular, YRNA fragments are the most abundant small RNA subtype, and are more abundant in NSCLC plasma EVs than healthy controls. Our analyses suggest that most small RNA types, including YRNA, tRNA, piRNA, rRNA, and snRNA fragments, have different expression and distribution signatures in EVs from NSCLC patients and healthy controls, and are likely to be involved in NSCLC pathogenesis. Further analysis of YRNA fragments indicates that the majority of upregulated YRNA fragments in NSCLC plasma EVs are hY4 RNA-derived fragments. Interestingly, we found that the *RNY4P7* gene on chromosome 2, corresponding to hY4rF, may not be a pseudogene. Besides, we found that hY4-derived fragments, hY4F and hY4rF, could inhibit NSCLC cell proliferation. Our analysis of miRNAs indicated that some miRNAs expressed in plasma EVs differ among ADC, SQCC, and CTRL groups. We further found that miR-451a, miR-122-5p, miR-20a-5p, miR-20b-5p, miR-30b-5p, and miR-665 are significantly upregulated in NSCLC EVs, suggesting that these miRNAs may serve as potential circulating biomarkers for the diagnosis of NSCLC. We also found that expression of miR-451a and miR-122-5p was significantly downregulated in NSCLC cell lysates, while significantly upregulated in NSCLC EVs, indicating that miR-451a and miR-122-5p may be selectively sorted into NSCLC EVs.

### Differentially expressed YRNA fragments in NSCLC EVs

We found that EV YRNA expression was significantly higher in NSCLC than in healthy individuals, suggesting that EV YRNA may be a potential biomarker for NSCLC diagnosis. YRNAs are 84–113 nt small non-coding RNAs with stem-loop structures that bind Ro60 and La proteins to form the Ro ribonucleoprotein complex [46]. There are four YRNAs in human: hY1, hY3, hY4, and hY5. YRNAs are upregulated in some human tumor tissues, and hY1 and hY3 are required for cell proliferation [47], suggesting that YRNAs are closely associated with carcinogenesis. YRNA fragments have also been found in EVs from both normal and cancer cells [45], as well as in human serum and plasma [48], suggesting a potential function in EV-mediated intercellular communication. Other studies have identified the presence of YRNA fragments in EVs from both breast cancer and normal breast cells, however there was no significant difference in YRNA expression between the two types of EVs [45]. Although it is generally acknowledged that YRNAs are clustered together at a single locus on human chromosome 7q36 [49], some studies show that YRNAs also have pseudogenes on other chromosomes in the human genome [35, 36]. A recent report showed that over 85% of YRNA-derived fragments in EVs from human breast cancer cells and non-tumor breast cells corresponded to hY4 RNA [45]. Similarly, we found that most of the YRNA fragments upregulated in plasma EVs of NSCLC patients were hY4 RNA fragments, and the *RNY4P7* gene on chromosome 2 may not be a hY4 RNA pseudogene. Additionally, we found that hY4-derived fragments (hY4F and hY4rF) were significantly down-regulated in NSCLC cells, and inhibited the proliferation of NSCLC cell A549, suggesting that hY4-derived fragments play important roles in NSCLC genesis and progress.

### Expression of other small RNAs in NSCLC EVs

We also found that other types of small RNAs in plasma EVs have different expression profiles among ADC, SQCC, and CTRL groups. For example, SQCC EVs had higher levels of tRNA fragments, and NSCLC EVs had lower levels of rRNA, piRNA, and snRNA fragments than in EVs from healthy controls. tRNA fragments are the cleavage forms of the mature tRNAs or tRNA precursors, and are heterogeneous in size, nucleotide composition, biogenesis, and function [50]. tRNA fragments are associated with breast cancer [51] and prostate cancer [52] progression, and may act as miRNA-like molecules and as post-transcriptional regulators [53]. Studies have reported that tRNA-derived fragments are present in EVs from human semen [54], plasma [32], and breast cancer cells [45], and the tRF-Leu-CAG tRNA fragment, which is upregulated in NSCLC and NSCLC cell lines, can promote cell proliferation [55]. Similarly, our studies suggest that EV tRNA fragments may be associated with NSCLC pathogenesis, particularly in SQCC.

In addition to tRNAs, our study also demonstrated that rRNA, snRNA, and piRNA have differential expression signatures in EVs from NSCLC patients compared to healthy controls, and therefore, may serve as potential biomarkers for NSCLC. snRNAs are a class of small non-coding RNAs (about 150 nt), that form the main component of the spliceosome complex [56]. Of interest, circulating U2 snRNA fragments can be a diagnostic biomarker for ovarian cancer [57], pancreatic cancer, and colorectal cancer [58]. piRNAs are 26–31 nt small non-coding RNA molecules that form RNA–protein complexes through interactions with piwi proteins [59]; they are involved in gene silencing [60] and epigenetics [61], and are biomarkers in various cancer types [62]. Multiple studies have identified piRNA in exosomes from human saliva [63] and plasma [32]. Although our study has identified small RNA species that are candidate biomarkers for NSCLC, further research is required to determine the specific sequences of these EV RNA species, and more importantly, to evaluate their functions and mechanisms in NSCLC pathogenesis.

### Differently expressed miRNAs in NSCLC EVs

Our miRNA expression analysis showed that the majority of EV miRNAs from the CTRL group have a lower expression than those from ADC and SQCC groups, and there is less diversity of EV miRNAs between ADC and CTRL than between SQCC and CTRL. The EV miRNA profiles also showed that the majority of miRNAs were not significantly differentially expressed, and about 10% of miRNAs were significantly upregulated in NSCLC plasma EVs, while only about 3% of miRNAs were significantly downregulated in NSCLC plasma EVs.

Our sequencing results also indicate that there is bigger difference of EV miRNA between SQCC and CTRL groups than between ADC and CTRL groups, which may represent a distinction between ADC and SQCC. Multiple studies have shown many differences between ADC and SQCC clinical and pathological characteristics [24], as well as genomic alteration [26] and gene expression profiles [25]. For example, recent studies on exome sequencing and copy number profiles of 1144 lung cancers demonstrated that somatic gene alterations, including gene mutations and recurrent somatic copy number alterations, are largely distinct in lung ADC and SQCC [26]. It has also been widely reported that miRNA expression profiles are very different between lung ADC and SQCC tumors [64]. Furthermore, recent studies on tumor-derived exosomal miRNAs have also demonstrated the exosomal miRNA expression variety between lung ADC and SQCC, and identified ADC- or SQCC-specific miRNAs for the diagnosis of NSCLC subtypes [29]. Overall, our EV miRNA profiles illustrate differences among lung ADC, SQCC, and healthy individuals, including distinctions between lung ADC and SQCC, consistent with previous reports.

Our qRT-PCR results showed that expression of miR-20a-5p, miR-20b-5p, miR-30b-5p, miR-451a, and miR-665 are significantly upregulated in NSCLC plasma EVs, consistent with our RNA-Seq results. The recent reports on deep sequencing of tumor-derived exosomal miRNA from NSCLC (16 lung ADC and 10 lung SQCC) clinical stage I patients have shown that miR-451a is significantly upregulated and miR-665 is significantly downregulated in lung SQCC exosomes, and expression of miR-20a-5p, miR-20b-5p, and miR-30b-5p is not significantly different between ADC and SQCC [29]. Our findings regarding the expression pattern of miR-20a-5p, miR-20b-5p, miR-30b-5p, and miR-665 differ from these previous reports, perhaps because samples in our study were from patients of various clinical stages, while the previous studies contained only samples from stage I patients. In fact, other studies have shown that miRNA expression profiles from different clinical stages are distinct [65, 66]; for example, expressions of miR-21 and miR-4257 are significantly upregulated in plasma exosomes from NSCLC patients of more advanced stage [67].

### Selective sorting of small RNAs into NSCLC EVs

Although most small RNAs are released into extracellular circulation in a non-selective manner [68], some specific small RNAs are selectively sorted into exosomes and other EVs [42, 69]. There are two possible routes for miRNA export via exosomes, an Ago-associated pathway and a RNA-binding protein-dependent pathway. For example, some proteins, such as SYNCRIP and hnRNPA2B1 [70], can guide the sorting of certain miRNAs into exosomes by binding to specific motifs. Other proteins, such as the RISC (RNA induced silence complex [RISC]) protein AGO2, may control incorporation of exosomal miRNAs in a miRNA sequence-independent manner [71]. Sheckman and his co-workers found that RNA-binding protein YBX1 binds to miR-223 and is required for its selective sorting into exosomes, however they failed to discover the recognition motif sequences, suggesting the possibility that the recognition motif for sorting into exosomes may be based on secondary rather than primary RNA structures [72]. Of interest, we found that the expression of miR-451a and miR-122-5p were significantly downregulated in NSCLC cell lysates, but significantly upregulated EVs of the same cells, indicating that miR-451a and miR-122-5p are likely to be selectively sorted into NSCLC EVs.

Both miR-122-5p [73–75] and miR-451a [76–79] are tumor suppressor miRNAs in various tumors [80]; in lung cancer, miR-122-5p inhibits metastasis and epithelial mesenchymal transition of NSCLC cells [81], while miR-451a inhibits proliferation of NSCLC cells, enhances NSCLC cell apoptosis, and suppresses lung tumor growth [40]. Similarly, it was reported that major vault protein mediates selective sorting of the tumor suppressor miR-193a into colon cancer cell exosomes, which paradoxically promotes cancer cell proliferation and tumor progression [10]. Therefore, the sorting of tumor suppressor miR-122-5p and miR-451a by NSCLC cells into EVs may indirectly play a role in promoting NSCLC progression.

Additionally, another recent report demonstrated that exosomal delivery of miR-122-5p suppresses glucose uptake by niche cells by downregulating the glycolytic enzyme pyruvate kinase, thus promoting tumor metastasis by increasing nutrient availability in the premetastatic niche [82]. And miR-451a has also been reported as a negative regulator of glucose production and glucose homeostasis by targeting glycerol kinase-mediated gluconeogenesis in normal hepatic cells [83]. Our KEGG pathway analysis showed that predicted targets of miR-122-5p and miR-451a are significantly enriched for the endocytic pathway, that is, miR-122-5p and miR-451a are very likely to regulate the endocytic pathway. Thus, NSCLC EVs delivery of miR-122-5p and miR-451a may also function to reprogram metabolism processes or interfere with endocytosis of a non-tumor cell, which conversely may provide a more appropriate environment for tumor progression and metastasis.

## Conclusions

Overall, we have illustrated the different expression signatures of various small RNA species in EVs from lung ADC, SQCC, and healthy controls. We first identified EV YRNA fragments as a novel candidate class of circulating biomarkers for NSCLC diagnosis, and found that hY4-derived fragments functioned as tumor suppressors in NSCLC. Additionally, we demonstrated that miR-451a and miR-122-5p may be selectively sorted into NSCLC EVs. Although our studies were properly designed, including the use of an appropriate sample number and multi-aspect validation with clinical samples and cultured cell lines, some deficiencies and limitations exist. For instance, the robustness of our small RNA sequencing results was not high enough, and many differentially expressed miRNAs identified by RNA-Seq were not consistent with the qRT-PCR validation results. Importantly, our studies have indicated a wider range of biological effects in NSCLC that may be mediated by EV small RNAs than previously known. Nevertheless, further thorough investigations of the function and mechanism of these EV small RNAs in tumorigenesis are urgently needed.

## Methods

### Cell culture

The human lung adenocarcinoma cell lines (A549, H157, H1975, H2228, H3122, H2030, and H1299), and human large cell lung cancer cell line H460 were cultured in RPMI-1640 medium (GIBCO); the human squamous cell lung carcinoma cells (SK-MES-1) were cultured in MEM medium (GIBCO); and the human normal embryonic lung fibroblast cell line IMR-90 was cultured in MEM medium supplemented with 10% FBS (GIBCO), GlutaMAX (GIBCO), MEM non-essential amino acids solution (GIBCO), sodium pyruvate (GIBCO), and penicillin–streptomycin solution. All cells were maintained in a humidified chamber at 37 °C in 5% CO_2_.

### Patient plasma samples

Plasma samples from lung ADC patients, SQCC patients, and healthy individuals were provided by Tongji Hospital and Hubei Cancer Hospital. The diagnosis of lung cancer subtypes was referred to 2015 World Health Organization classification of lung tumors. And healthy individuals without lung cancer or any other diseases were selected as the control group. Blood was collected and dispensed into an EDTA anticoagulation tube, then mixed gently to ensure exposure to the EDTA-coated walls. Plasma was separated by centrifugation at 5000 rpm for 5 min at 4 °C. The clear supernatant was transferred to a labeled tube, and stored at − 80 °C. This study was approved by ethics committees of Tongji Hospital and Hubei Cancer Hospital, and all methods were carried out in accordance with relevant guidelines and regulations. All donors gave permission and written informed consent was obtained from all participants.

### Preparation of EV-free FBS

Sixty milliliters of fetal bovine serum (FBS; GIBCO) was added to 11 ml ultracentrifuge tubes with an adaptor, placed in a Type P40ST-2079 rotor (Hitachi), and spun in a Hitachi CP80WX ultracentrifuge at 100,000*g* at 4 °C for 16 h. The supernatant of each tube was then transferred to a 50 ml tube and stored at − 20 °C for future use.

### EV isolation from plasma

Extracellular vesicles from plasma were isolated using Ribo™ Exosome Isolation Reagent (CAT. NO. C10110-2, Ribobio, China) according to the manufacturer’s protocol. First, plasma samples were centrifuged at 2000*g* at room temperature for 20 min to remove residual cells and debris, and then the supernatant was transferred to a new tube and centrifuged at 10,000*g* at room temperature for 20 min. The supernatant was transferred to a new tube and mixed adequately with 1/3 volume isolation reagent, and the mixture was placed at 4 °C for 30 min and centrifuged at 15,000*g* at 4 °C for 2 min. Finally, the supernatant was removed and the EV pellet was recovered by re-suspending in phosphate-buffered saline (PBS).

### EV isolation from cell culture medium

After incubating cells with medium containing EV-free FBS for 48 h, EVs from cell culture media were isolated using Ribo™ Exosome Isolation Reagent (CAT. NO. C10130-2, Ribobio, China) according to the manufacturer’s protocol. First, cell culture medium was centrifuged at 2000*g* at room temperature for 30 min to remove residual cells and debris, and then the supernatant was transferred to a new tube and mixed with 1/3 volume isolation reagent. Next, the mixture was placed at 4 °C overnight, and then centrifuged at 1500*g* at 4 °C for 30 min. Finally, the supernatant was removed and the EV pellet was recovered by re-suspending in PBS.

### Western blot analysis

Extracellular vesicle protein was prepared by re-suspending EV pellets in RIPA buffer supplemented with protease inhibitor (Roche). Twenty micrograms of protein from each sample was separated on a 10% SDS-PAGE gel in parallel with a protein marker (Thermo). Proteins were transferred to a polyvinylidene fluoride (PVDF) membrane, and blocked with 5% skim milk dissolved in Tween/Tris buffered saline (TTBS) for 1 h at room temperature. Membranes were then incubated with primary antibodies against TSG101 (ProteinTech) or CD9 (ProteinTech) at a dilution of 1:5000 with 3% skim milk diluted in TTBS for 1 h at room temperature. After washing 6 times with TTBS, membranes were incubated with horseradish peroxidase conjugated goat anti-rabbit IgG secondary antibodies (Santa Cruz Biotechnology) at a dilution of 1:10,000 in 3% skim milk dissolved in TTBS for 1 h at room temperature. After washing 6 times with TTBS, membranes were covered with ECL western blot substrate solution and visualized by exposing to film and developing in a film processor.

### Transmission electron microscopy (TEM)

The EV pellets were re-suspended in 100 µl of 2% paraformaldehyde, and 5 µl was placed on Formvar–carbon-coated electron microscopy grids and adsorbed by the membrane for 20 min in a dry environment. After washing the samples with several drops of PBS on a sheet of Parafilm, samples were incubated on drops of buffered 1% glutaraldehyde for 5 min and then washed several times on drops of distilled water. Next, samples were negatively stained on drops of 4% uranyl acetate for 10 min on ice. Then, the grids were removed and excess fluid was blotted with filter paper, so that a thin film was left behind over the EV side of the grid. The samples were allowed to dry, and observed using a JEM-1400plus transmission electron microscope (JEOL) at an accelerating voltage of 80 kV.

### Flow cytometry analysis of exosomal marker proteins

Isolated EV pellets were diluted in PBS, then incubated with FITC-conjugated anti-CD63 or anti-CD81 antibodies (Abcam, US) diluted in PBS/0.5% BSA for 1 h at 37 °C. The EV samples were analyzed with a Cytoflex flow cytometer (Beckman, US), and IgG incubated samples were used as a negative control.

### Nanoparticle tracking analysis

Isolated EV pellets were diluted in PBS and analyzed using the Nanosight NS300 system (Malvern Instruments, UK). The particle size distribution was calculated using Nanosight Tracking Analysis software. Size distribution profiles were averaged across three replicates or each sample to derive the representative size distribution profiles.

### MTT assay

To assess cell proliferation, cells were transiently transfected with miRNAs mimics (Ribobio, China) for 24 h using lipofectamine 2000 (Thermo, US), and then seeded in 96-well culture plates at a concentration of 6000 cells per well, with each group comprised of six replicates. The cells were incubated with 3-(4,5-dimethylthiazol-2-yl)-2,5-diphenyl tetrazolium bromide (MTT) (Sigma, US) for 4 h at 37 °C, and then the supernatant was removed and dimethyl sulfoxide was added. Absorbance was measured at 570 nm with microplate reader (BioTek, US), and above tests were performed every 24 h for consecutive days.

### Colony formation assay

After transfected for 24 h, cells are seeded into 6-well culture plates in dilutions of 200 cells per well to form colonies in 9 days. Colonies are fixed with 4% (v/v) glutaraldehyde for 15 min, stained with 1% (w/v) crystal violet for 20 min, and counted using a stereomicroscope.

### RNA extraction

Isolation of total RNA from cells or EVs was performed with Trizol reagent (Invitrogen, Life Technologies) and the Direct-zol™ RNA MiniPrep kit (Zymo Research Corp.) according to the manufacturer’s protocol. RNA concentration was quantitated using a NanoDrop 2000 Spectrophotometer (Thermo Scientific).

### Quantitative real-time reverse transcription PCR (qRT-PCR)

Spike-in control cel-miR-39 mimic (miRNeasy Serum/Plasma Spike-In Control, Qiagen) was added to total RNA samples as internal control, and the mixed RNA was reverse-transcribed to cDNA using a reverse transcriptase kit (miScript II RT Kit, Qiagen). SYBR-based qRT-PCR was performed on an ABI7500 real-time PCR amplifier (Applied Biosystems) using specific primers (miScript primer Assay, Qiagen) and a universal PCR kit (miScript SYBR Green, Qiagen) according to the manufacturer’s protocol. For hY4 RNA fragments detection, U6 snRNA was used as internal control. Amplifying conditions were as follows: 95 °C for 15 min, followed by 40 cycles of 94 °C for 15 s, 55 °C for 30 s, and 70 °C for 34 s [84, 85]. Gene expression data were normalized to internal control expression, and the relative expression was determined as 2^−Δ Ct^, where Δ Ct = Ct (target gene) − Ct (internal control).

### cDNA library generation and high-throughput sequencing

Sequencing libraries were generated as follows: following isolation of total RNA from plasma EVs, RNA samples were analyzed with an Agilent 2100 Bioanalyzer (Agilent) using a total RNA nanochip, then the small RNA fractions (< 60 nt) were separated by electrophoresis. Next, small RNA fragments were ligated to 5′-adapter and 3′-adapters, and then reverse-transcribed and PCR amplified, with indexes included in the PCR reaction. PCR products were run on a 6% polyacrylamide gel and selected to include RNAs with an insert size < 51 nt. The DNA-containing index was eluted from the gel, precipitated, and dissolved in nuclease-free water. Small RNA inserts were sequenced using a HiSeq 2500 high-throughput sequencing system (Illumina).

### Sequencing data processing and analysis

To get clean reads, the adaptor sequences, contaminated reads, low quality reads, less than 17 nt reads, and poly-A/T/C/G/N repeat sequences were removed. Next, the clean reads (17–45 nt) were mapped to the human genome database using Burrows–Wheeler Aligner software [86]. Uniquely aligned reads were categorized and annotated to known non-coding small RNA transcripts using Rfam version 11.0 [87] for YRNA, rRNA, snRNA, snoRNA, and tRNA. miRBase version 21 [88] was used for miRNA, and piRNABank [89] was used for piRNA.

Next, the quantity of small RNA in each sample was obtained and normalized as RPM (number of mapped reads per million clean reads). The clean reads of miRNA, containing the entire reference sequence from the miRBase database and a length difference within 4 nt, were confirmed, as well as miRNA generated from the same end of the same precursor and a length discrepancy below 3 nt. Considering sequencing error, reads with less than ten copies were removed. The sequence length distribution and percentage of different small RNA species were also analyzed with the statistical data of reads. The distribution of small RNA across the whole human genome was performed using a R language package RCircos [90]. The cumulative frequency distribution was performed using GraphPad Prism 5, and the relative frequency is shown as a fraction. The correlation analysis was also determined using GraphPad Prism 5 with a Gaussian distribution.

Significant differences in miRNA expression were determined using R package edgeR [91], with thresholds of a P value < 0.05, a false discovery rate (FDR) < 0.05, and a |log2 (Fold Change)| ≥ 1. Scatter plots and MA plots of miRNA expression differences were generated using R package edgeR. The heatmap of miRNA expression differences was plotted using function heatmap.2; in R package gplots, and miRNAs with extremely low abundance (less than 1 RPM) in all groups were removed. Considering that RNA-Seq produces a wide range of read counts per gene, and genes with a low coverage of reads can produce artificially high fold-change values [92], RPM was added with a moderate cutoff of 0.1 and transformed as log2 for drawing heatmaps.

### Gene ontology (GO) and KEGG enrichment analysis of predicted miRNA targets

miRNA targets were predicted with default parameters using starBase version 2.0, a database of predicted miRNA targets gathered from targetScan, picTar, RNA22, PITA, and miRanda databases [93]. The GO enrichment analysis of miRNA targets was performed with the BinGO plugin [94] for Cytoscape software [95] version 3.4.0 using whole annotation as a reference set; hypergeometric test, false discovery rate (FDR) correction, P < 0.05 significance level and GO BP (Biological Process) ontology files were selected. For KEGG pathway enrichment analysis, the DAVID online tool (https://david.ncifcrf.gov) [96] was used with Fisher’s exact test, FDR correction, and P < 0.05 significance level.

## Additional file

**Additional file 1.** Additional Figures S1–S7 and Tables S1–S5.
